# Low Intraprostatic DHT Promotes the Infiltration of CD8+ T Cells in BPH Tissues *via* Modulation of CCL5 Secretion

**DOI:** 10.1155/2014/397815

**Published:** 2014-04-06

**Authors:** Yu Fan, Shuai Hu, Jie Liu, Fei Xiao, Xin Li, Wei Yu, Yun Cui, Mengkui Sun, Tianjing Lv, Qun He, Jie Jin

**Affiliations:** ^1^Department of Urology, Peking University First Hospital and Institute of Urology, Peking University, Beijing 100034, China; ^2^National Research Center for Genitourinary Oncology, Beijing, China; ^3^Department of Surgery, China-Japanese Friendship Hospital, Beijing, China

## Abstract

Clinical studies suggested thatandrogen might be associated with infiltrating T cells in prostate of benign prostatic hyperplasia (BPH) patients, but detail of T-cell subset and mechanism still remained unclear. The present study tested the hypothesis that intraprostatic 5**α**-dihydrotestosterone (DHT) exerts effects on T cells recruitment by BPH epithelial cells. Prostate tissues from 64 cases of BPH patients after transurethral resection of prostate (TURP) were divided into 2 groups: (1) no medication history; (2) administration of 5**α**-reductase type II inhibitor-finasteride 5 mg daily for at least 6 months before surgery. Group 2 presented significantly higher CD8+ T cells infiltration than group 1, but no changes in CD4+ T cells (immunohistochemistry and flow cytometry). *In vitro* study more CD8+ T cell migrated to the prostate tissue lysates from group 2 and BPH-1 cells in low DHT condition. Transcription of chemokine (C-C motif) Ligand 5 (CCL5) mRNA in BPH-1 cells and chemokine (C-C motif) receptor 5 (CCR5) mRNA in CD8+ T cells were upregulated in low DHT condition (q-PCR). CCL5 expression was also identified to be higher in group 2 prostate tissues by IHC. This study suggested that intraprostatic DHT may participate in regulating inflammatory response which was induced by human prostatic epithelial cell, via modulating CCL5 secretion.

## 1. Introduction

Benign prostatic hyperplasia (BPH) is one of the most common chronic diseases in aging men [1]. The most potent androgen in men, 5*α*-dihydrotestosterone (DHT), is widely accepted to be more essential than testosterone for prostatic epithelial cell proliferation and function [2]. It is believed to be largely converted from testosterone in prostate by the action of 5*α*-reductase enzyme [3]. So the mainstay of therapies is the 5*α*-reductase inhibitors, which regulate the levels of intraprostatic DHT. Finasteride, a competitive inhibitor of 5*α*-reductase-type II with relatively low affinity for type I [4], has been one of the most commonly prescribed drugs for the management of BPH. It markedly reduces intraprostatic DHT concentration [5]. 5*α*-reductase inhibitors (finasteride) exert a strong apoptotic effect on the DHT-dependent epithelium, but in some BPH patients finasteride fails to control symptoms.

To date, chronic inflammation has been recognized as another key player in the BPH pathogenesis and progression [6]. Meanwhile, many studies have showed that the majority of lymphocytes in BPH tissue were T-lymphocytes [7, 8]; infiltration of chronically T lymphocytes and secretion of inflammatory cytokines with the prostatic gland are considered a determinant factor in BPH pathogenesis and progression [9]. Unfortunately, most of the recent studies focused on the proinflammatory cytokines secreted by T-lymphocytes and BPH cells [10]. The causes for T-cell infiltration and immune dysregulation in the prostate remain subjects of debate.

The most important potential cause for immune response in prostate is the prostatic microenvironment [11]. BPH epithelial cells, an important component of prostatic microenvironment, are suggested as a key role of the induction of immune-mediated inflammatory processes [12]. On the other hand, intraprostatic DHT could affect function of epithelial cell directly [13]. Accordingly, it suggests some linkages between inflammation and intraprostatic DHT. Vignozzi et al. (2012) [14] reported that intraprostatic testosterone plays a protective role in metabolic syndrome-associated prostate inflammation in rabbit model. From a pathophysiological standpoint, some studies showed correlation between DHT level and inflammation [15], but the detail of T cell subsets infiltration influenced by BPH epithelial cells and intraprostatic DHT still remained largely unresolved.

In the present work, we focused on the relationship between intraprostatic DHT level, BPH epithelial cells, and T-cell infiltration. We further elucidated the chemokine changes in different level of intraprostatic DHT.

## 2. Materials and Methods

### 2.1. Materials

#### 2.1.1. Patients

The patients were selected by considering medication duration from the medical records of 726 patients who underwent transurethral resection of the prostate (TURP) between January 2008 and December 2011 in Peking University First Hospital. According to the medication history, prostate tissue was obtained from 64 prostatic hyperplasia patients by transurethral resection. Patients were divided into two groups: group 1 consisted of 28 patients who had been medicated neither with *α*-adrenergic blocker nor with 5*α*-reductase inhibitor; group 2 consisted of 36 patients treated with finasteride 5 mg daily for longer than six months before surgery. All included samples were pathologically confirmed as benign prostatic hyperplasia without prostate cancer or prostatic intraepithelial neoplasia (PIN). And the patients who had urinary tract infection or previous prostate-related surgery or were treated with urinary catheter were excluded from the study.

#### 2.1.2. Reagents and Antibodies

Monoclonal anti-CCL5 antibodies and the recombinant protein IgG were purchased from R&D systems (MAB678, MAB002, Minneapolis, MN, USA), and 500 *μ*g/mL stock was reconstituted in phosphate buffered saline (PBS). For anti-CCL5 treatment, stocks were adjusted to a final concentration of 6 *μ*g/mL. Ficoll-Paque was purchased from Amersham Pharmacia Biotech (17144002, Piscataway, NJ, USA). Collagenase D was purchased from Roche Diagnostic (100 mg, 11088858001 Indianapolis, IN, USA) and was adjusted to a final concentration of 1 mg/mL to use. Antibodies used for flow cytometry included PE-Cy 7-conjugated mouse anti-human CD3 antibody (341091), fluorescein isothiocyanate (FITC)-conjugated mouse anti-human CD4 antibody (340133), phycoerythrin (PE)-conjugated mouse anti-human CD8 antibody (340046), PE-Cy 7-conjugated mouse IgG1, *κ* isotype (555872), FITC-conjugated mouse IgG1 *κ* isotype (555909), or PE-conjugated mouse IgG1*κ* isotype (554680). All these FACs antibodies were purchased from BD Biosciences (NJ, USA). Antibodies used for immunohistochemistry included Rabbit anti-CD4(+) (dilution 1 : 50, ab133616, Abcam, Cambridge, UK), anti-CD8(+) (dilution 1 : 50, RM-9116-S1, Thermo Fisher Scientific, Cheshire, UK), and the rabbit anti-CCL5 (+) (2 *μ*g/mL, ab9679, Abcam, Cambridge, UK).

### 2.2. Experimental Procedures

#### 2.2.1. Blood Sample Preparation

Blood samples were collected in sterile heparinized containers from 6 health persons at 10 mL per tube. Purification of human Peripheral blood mononuclear cells was performed according to some previous publications [16]. Peripheral blood mononuclear cells (PBMC) were isolated by Ficoll density gradient and blood was centrifuged for 20 min at 2000 ×g. The cells were washed twice in phosphate buffered saline (PBS, pH 7.2) without calcium and magnesium and resuspended in X-VIVO 15 medium (04-418Q, Lonza, NJ, USA) for further analysis.

#### 2.2.2. Prostate Tissue Preparation

Prostate tissue preparation was performed as the publication description [17]. One part of fresh prostate tissue which was got from surgery was placed in a solution of 1 mg/mL Collagenase D (Roche) in RPMI 1640 media containing 10% FBS with DNase I (20 *μ*g/mL; Sigma-Aldrich, St. Louis, MO, USA). Tissue was minced at about 1 mm^3^ and placed at 37°C for 1 hr for digestion, followed by passing through a 70 *μ*m filter. Then the lymphocytes were isolated by Ficoll density gradient. Then the tissue lysates were filtered through 0.22 *μ*m filter and 6 cases of these tissue lysates from each 2 groups were chosen randomly for the further migration assay. The other part of fresh tissue was formalin-fixed for the IHC staining.

#### 2.2.3. Cell Culture

The BPH epithelial cell-line, BPH-1, was purchased from KeyGen Biotech Co., Ltd (KG1008, NJ, China) and the predominantly CD8(+) T-lymphocytic cell-line, Molt-3 [18], was purchased from the American Type Culture Collection (CRL-1552, Rockville, MD, USA) and grown in RPMI-1640 media containing 1% penicillin and streptomycin, supplemented with 10% fetal bovine serum (FBS). All cell lines were cultured in a 5% (v/v) CO_2_ humidified incubator at 37°C. BPH-1 cells of charcoal medium group were treated with 10% charcoal treated fetal calf serum (SH30068.03, Hyclone, South Logan UT, USA) for 2 days, then collected supernatant and BPH-1 cells were harvested for the further experiment. Peripheral blood mononuclear cells (PBMC) that were isolated from human blood were grown in X-VIVO 15 medium (04-418Q, Lonza, NJ, USA) and all the further experiment would be finished in 2 days.

### 2.3. Methods

#### 2.3.1. Immunohistochemistry

To determine the expression of CD4 and CD8 on the cell surface and intracellular CCL5 in BPH tissues, the specimens from TUR-P surgery were fixed in 4% buffered formalin overnight at 4°C and then dehydrated in an ascending ethanol series, routinely embedded in paraffin, and sectioned at 3 *μ*m. After conventional deparaffinization, hydration, and antigen retrieve, endogenous peroxidase was inactivated by 3% hydrogen peroxide. The primary antibodies of the rabbit anti-CD4(+) (dilution 1 : 50, Abcam), anti-CD8(+) (dilution 1 : 50, Thermo), and the rabbit anti-CCL5 (+) (2 *μ*g/mL, Abcam) were used for incubation at 4°C overnight. After washings with phosphate-buffered saline (PBS), the primary antibody was recognized by the biotinylated secondary antibody (PK-4001, Vector Labs, Burlingame, CA, USA) at room temperature for 30 min and visualized by VECTASTAIN ABC peroxidase system and peroxidase substrate DAB kit (SK-4100, Vector Labs).

The max density of CD4 positive or CD8 positive cell was defined as ratio between the maximum positive cell number and all of nuclear cell number under 100x field. And these indexes were average value by two operators who are blind to each other and were calculated with Image-Pro Plus 6.0 software (Media Cybernetics, Rockville, MD, USA).

Proteins expression of CCL5 was assessed semiquantitatively and a 4-tiered system (0 negative, 1 weak, 2 moderate, and 3 strong) was used. Two pathologists evaluated the stain strength and the final result was the average of their scores.

#### 2.3.2. Flow Cytometry

To further study the T-cell subpopulation infiltration among the total T cell, the isolated lymphocytes from prostate tissues were analyzed by two-color flow cytometry for phenotypic characterization of T cells according to manufacturer's procedure. Cells were resuspended in staining buffer (PBS containing 1% fetal bovine serum) and stained for 30 min at 4°C with anti-CD3 PE-Cy 7 and anti-CD4 FITC or anti-CD8 PE and their isotype control antibodies (both from BD Biosciences). Flow cytometry was done on a Becton Dickinson LSRII (BD Biosciences); 1 × 10^5^ cells were acquired and data were analyzed using Flow Jo software (BD Biosciences). Then the average percentage of CD4 positive or C8 positive among total T cell in each group was calculated. At the same time the CD8+ T cells from blood of healthy persons were also harvested by the same method.

#### 2.3.3. Quantitative PCR

Total RNA was extracted from each cell line using Trizol (15596-018, Invitrogen, Grand Island, NY, USA). According to manufacturer's protocol, cDNA was synthesized from 1 *μ*g RNA, using a High capacity cDNA reverse transcription kit (4368813, Applied Biosystem, CA, USA). The standard PCR conditions included 2 minutes at 50°C and 10 min at 95°C followed by 40 cycles of extension at 95°C for 15 seconds and one minute at 60°C. Threshold lines were automatically adjusted to intersect amplification lines in the linear portion of the amplification curves and cycle to threshold (Ct) were recorded automatically. Data were normalized with GAPDH mRNA transcription (housekeeping gene) and the fold change in gene expression relative to normal was calculated using the ddCt method. The sequences of the gene primers are designed by Primer Premier 5 software (Premier Biosoft International, Palo Alto, CA, USA) and are shown in Table 1.

#### 2.3.4. Migration Assay

To detect the recruitment of CD8+ T cell by tissue lysates and BPH-1 cells, the tissue lysates or 1 × 10^5^ of BPH-1 cells of different treatment were plated into the lower chamber of the transwells with 5 *μ*M pore polycarbonate membrane inserts (3421 Corning, MA, USA). 1 × 10^5^ of CD8+ T cells isolated by flow cytometry from 6 healthy persons and Molt-3 cells were plated onto the upper chamber. After 6 hrs, the cells migrated into the lower chamber media were collected and counted by the Bio-Rad TC10 automatic cell counter. Each sample was assayed in triplicate and each case was repeated twice.

#### 2.3.5. Statistical Analysis

Statistical analyses for continuous variables involved paired *t*-test with SPSS 17.0 (SPSS Inc., Chicago, IL, USA). Data were also analyzed through one-way ANOVA coupled with the Newman-Keuls test and Mann-Whitney test. *P* < 0.05 was considered statistically significant.

## 3. Results

### 3.1. The Influence of Finasteride Treatment on T Cell Population Infiltrating in BPH Prostate Tissue

**Figure 1:**
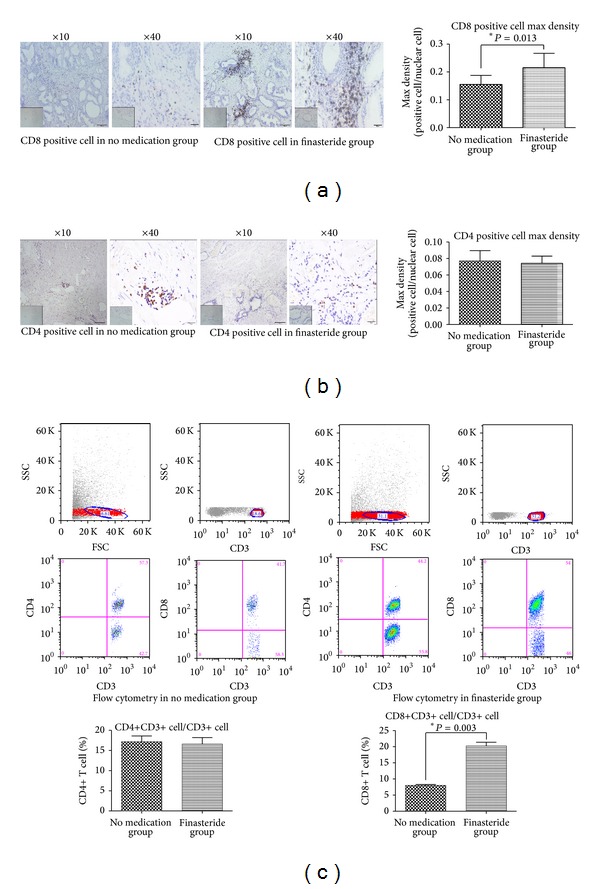
The T-cell population infiltrating prostate tissue with/without finasteride treatment. (a) CD8 was stained from no medication group and finasteride group; scale bar: 100 *μ*m and 20 *μ*m. Negative controls were showed in the bottom left corner, respectively. Data presented as the percentage of CD8+ T-cell number in all of nuclear cell number (mean ± SEM); *P = 0.013 (*t*-test). (b) Immunohistochemistry staining for CD4+ T infiltration. Magnification and negative control are the same as mentioned before. (c) Flow cytometry assay. Data presented as the rate of CD8+ or CD4+ T cells in total T cell (mean ± SEM). *P = 0.003 in the right column, (*t*-test).

We detected T-cell population infiltration between prostate tissue with/ without finasteride treatment [19]. Firstly, the immunohistochemical analysis using anti-CD4 and CD8 antibody showed that CD8+ T cells were identified surrounding the epithelium area, but CD4+ T cells in stromal area (Figures 1(a) and 1(b)).

Furthermore, as shown in Figure 1(a), the max densities of CD8+ T cells infiltrated in the finasteride group and in the no medication group were 0.23 ± 0.06, 0.14 ± 0.04, and they were significantly higher in the finasteride group than in the no medication group (*P* = 0.013). However, CD4+ T cells infiltration showed no difference (Figure 1(b)). Then flow cytometry data was consistent with the IHC staining. The tissues of group 2 presented a significantly higher percentage of CD8 positive cells among all total T-lymphocytes than tissues of group 1 (21.36% versus 8.78%, Figure 1(c)).

### 3.2. The CD8+ T Cells Migration* In Vitro*

**Figure 2:**
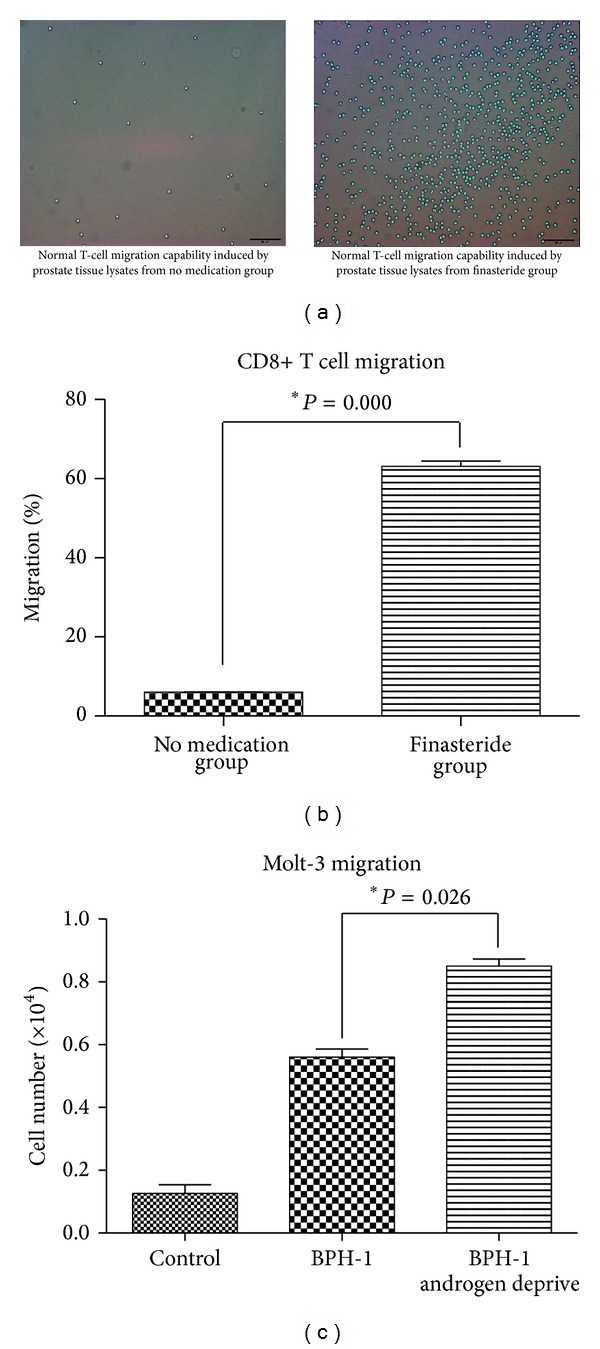
CD8+ T cells migration* in vitro*. (a) CD8+ T cell migrated to lower chamber after 6 hrs. Scale bar: 50 *μ*m. (b) Data showed as the percentage of migrated cell number in total cell number (mean ± SEM). *P < 0.000. (*t*-test). (c) The migration of molt-3 cells to BPH-1 cells with/without charcoal medium treatment in the lower chamber. Data presented as the average cell numbers (mean ± SEM). *P = 0.026. (ANOVA and Newman-Keuls test).

To study the potential cross talk between infiltrating CD8+ T cell and prostate epithelial cells as seen in BPH specimens (Figure 1), we established a coculture model for CD8+ T cell migration assay. As shown in Figures 2(a) and 2(b), more than 60% of CD8+ T from blood of health persons migrated to the prostate tissue lysates from the finasteride group. It was significantly higher than no medication group (64.02% versus 10.31%).

This data was then confirmed in BPH epithelial cell-line. As shown in Figure 2(c), BPH-1 cells which were pretreated with charcoal medium had more capability to recruit Molt-3 cells (*P* = 0.026).

### 3.3. Induction of Chemokines in BPH-1 Cells Stimulated by Changes of DHT Level

**Figure 3:**
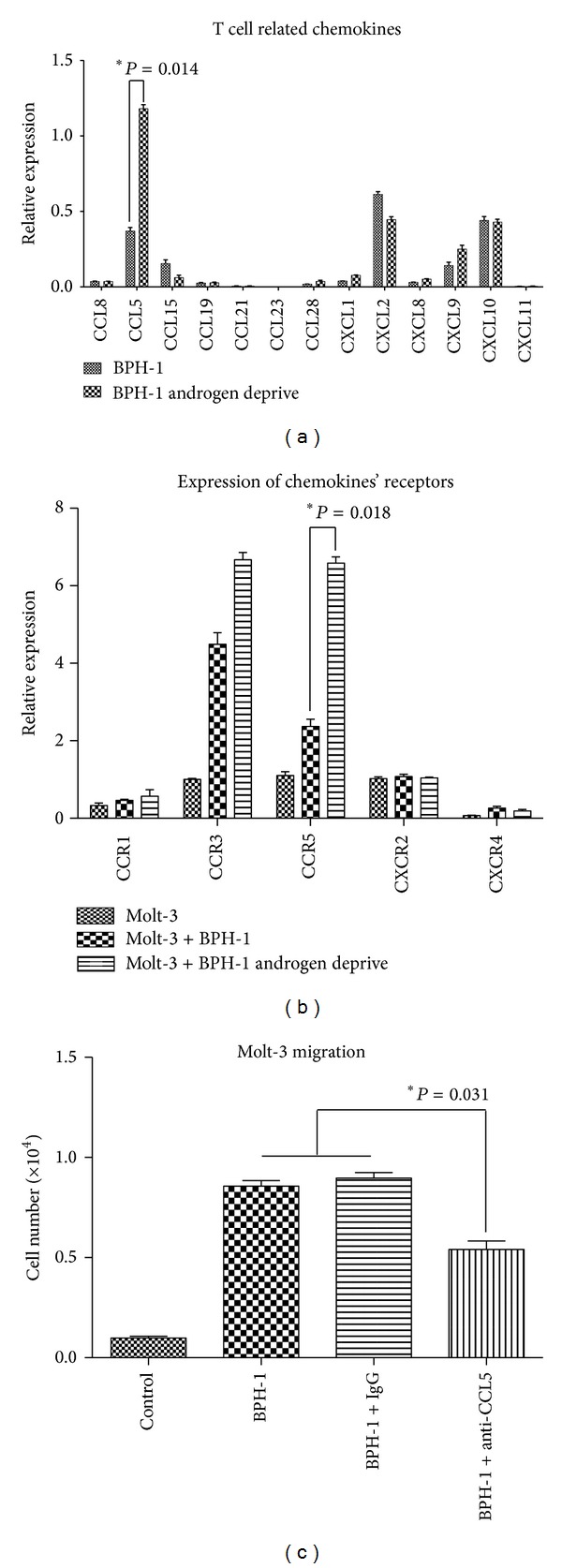
Induction of chemokines in BPH-1 cells stimulated by changes of DHT level. (a) Q-PCR screening of a panel of cytokine factors that could be responsible for BPH-1 cell promoted T-cell migration. Compared to the BPH-1 cells cultured with normal medium, mRNA transcription of CCL5 was upregulated in BPH-1 cells with charcoal medium treatment; *P = 0.014 (*t*-test). (b) Transcription of chemokine related receptors mRNA was detected by q-PCR. *P = 0.018 (ANOVA and Newman-Keuls test). (c) The interruption assay by adding CCL5 neutralizing antibody in above migration system. Data presented as the average cell numbers in lower chamber (mean ± SEM). *P = 0.031 (ANOVA and Newman-Keuls test).

The q-PCR was used to assay for the most reported chemokines that are related to attracting T cells [20, 21] from BPH-1 cells with normal versus charcoal medium. The transcription of CCL5 mRNA in BPH-1 cells was higher in lower DHT condition (1.18 ± 0.02) than those in normal condition (0.37 ± 0.05) (Figure 3(a)). In addition, mRNA level of CCR5 was also upregulated nearly 3-fold in Molt-3 cells after coculture with BPH-1 cells in charcoal medium as shown in Figure 3(b).

Next, interruption assay was detected by using CCL5 neutralizing antibody in the migration system. It was shown that blocking CCL5 led to significantly suppressing the Molt-3 cells migration toward BPH-1 cells in low DHT condition Figure 3(c).

### 3.4. CCL5 Expression in Clinical Samples with/without Finasteride Treatment

**Figure 4:**
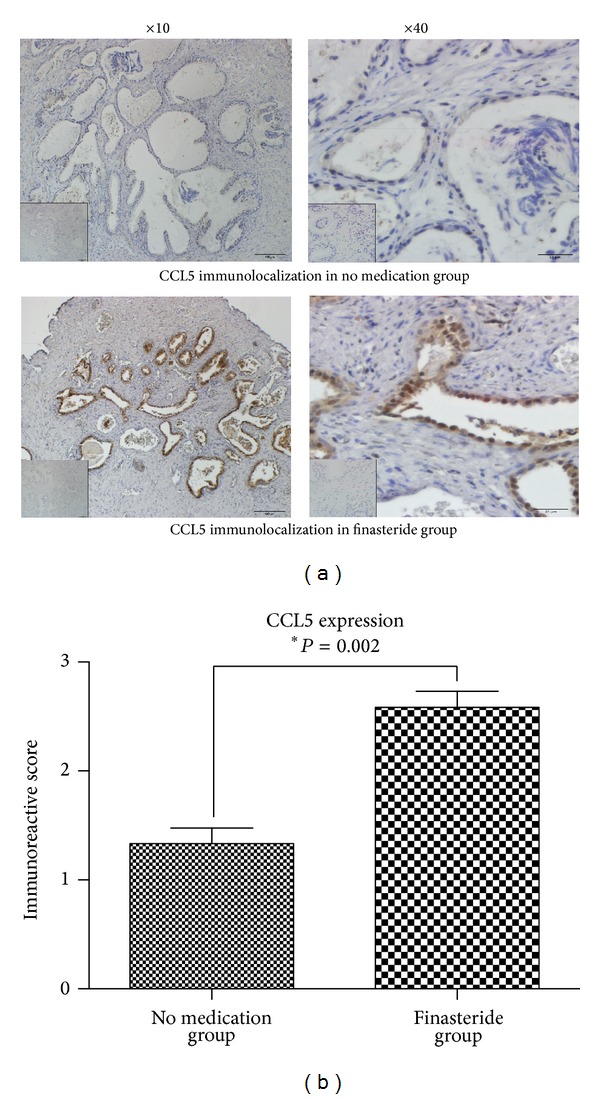
CCL5 immunolocalization in prostate tissue samples by IHC. (a) CCL5 was stained from no medication group and finasteride group. Magnification and negative control are the same as mentioned before. (b) The average immunoreactive score of CCL5 in different group was quantified (mean ± SEM). *P = 0.002 (Mann-Whitney test).

The CCL5 expression was investigated in above BPH patients by IHC staining as shown in Figure 4. The results showed that CCL5 expression located in the epithelial area. Meanwhile, immunoreactive score was higher in the finasteride treatment group (2.79 ± 0.26), compared to the no medication group (1.41 ± 0.28).

## 4. Discussion

At present, so many studies have shown the role of chronic inflammation in BPH development. Cytokines, growth factors like IL6, IL8, IFN-r produced by T-lymphocytes, and BPH cells are involved in altering tissue remodeling and hyperplastic growth at each stage of BPH [2]. However, few literatures focused on the aetiology of BPH chronic inflammation. Potential causes include infectious agents, exposure to other environmental and dietary factors, and hormonal and metabolic derangements [22]. In this study, we aimed to dissect the induction of immune response by prostatic environmental factors.

It is reported that prostatic immune inflammatory cells consist of 70% T lymphocytes, 15% B-lymphocytes, and 15% macrophages, as well as mast cells [23] (and our unshown data). Hence, in the present study, we focused on the T-cell subpopulation. To the best of our knowledge, 6 months finasteride treatment means low intraprostatic DHT level in these patients [24]. The IHC and flow cytometry results showed that finasteride treatment could lead to more infiltration of CD8+ T cells but not CD4+ T cells.

The IHC results in this study showed CD8+ T cells localized surrounding epithelial area in BPH tissue. BPH epithelial is an important component of microenvironment in the prostate tissue. It can acquire the ability to express class II MHC molecules [25]. BPH epithelial cells have been previously described to act as a key cell, indicating their potential role in inducing and sustaining an autoimmune response within the prostatic gland [26]. To better investigate whether androgens could directly suppress T-cell infiltration, we performed migration assay by using BPH-1 cells and Molt-3 cells* in vitro* studies with/ without low androgen condition. Data* in vitro* were consistent with* in vivo* IHC staining.

Results in this study demonstrated that intraprostatic DHT has strong immune suppressive effects on CD8+ T cell infiltration induced by BPH epithelial cells. Some other groups have also discussed the similar study. Vignozzi et al. highlighted that DHT exerts an immune regulatory role on human prostatic stromal cells, inhibiting their potential to actively induce and/or sustain autoimmune and inflammatory responses [27]. Park and Shim found that finasteride might interfere with the anti-inflammatory reaction induced by doxazosin in combination of doxazosin and finasteride treatment [28]. Here we detected more patients and identified the T cell subsets which infiltrated in the prostatic tissue after finasteride treatment. Importantly, we also tried to find the key chemokine.

To further dissect how BPH-1 recruited more Molt-3 cells, we applied q-PCR to examine the expression of T cell related chemokines in BPH-1 cells at low DHT level. We found that CCL5 which functions as a chemokine playing a critical role in the recruitment of T cells [29] was expressed significantly higher in BPH-1 cells with androgen deprivation. We also found that CCR5 which is the natural CCL5 coreceptor [30] was upregulated in Molt-3 cells after coculture with BPH-1 cell in low androgen condition.

Then CCL5 expression was identified in the clinical sample mentioned above. Higher expression of CCL5 is showed in BPH tissues after finasteride treatment by immunohistochemistry. Importantly, the CCL5 was localized surrounding the epithelial area. In normal and BPH prostate tissue, infiltrating CD8+ T cells are mainly localized around epithelial ducts [31, 32]. Herein, the CCL5 expression was consistent with the distribution of CD8+ T cells. It suggested that CCL5 may be the key chemokine which secreted by BPH epithelial cells to recruit CD8+ T cells after anti-DHT therapy.

**Figure 5:**
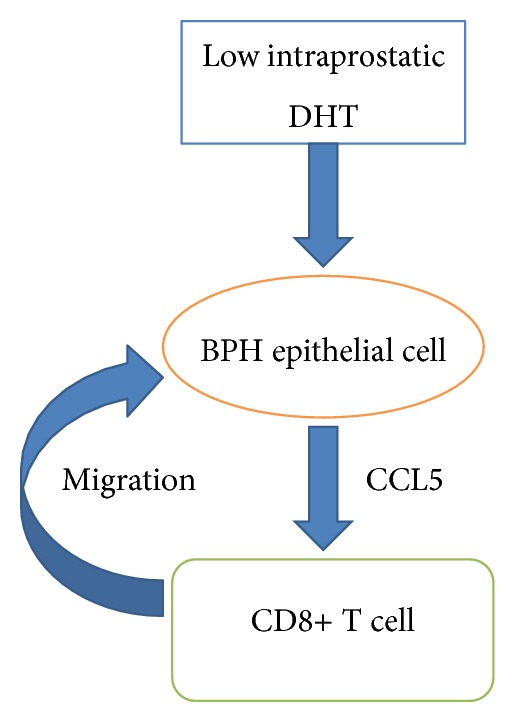
Mechanism and regulatory pathway of low intraprostatic DHT-promoted CD8+ T cell infiltration in BPH prostate tissue. Low intraprostatic DHT level could promote BPH epithelial cells to recruit more CD8+ T cells via upregulation of CCL5 mRNA transcription.

In conclusion, the most striking finding of the present study is that DHT exerts an immune regulatory role on human prostate epithelial cell, inhibiting their potential to actively induce inflammatory responses, and CCL5 which secreted by prostate epithelial cell is the key chemokine in this progression (Figure 5). Alternative anti-DHT therapy could lead to increased inflammation in prostatic tissue. This is might be a cause for treatment failure of 5*α*-reductase inhibitors. Anti-inflammation furthermore* via* targeting CCL5 combined with anti-DHT therapy in patients with BPH may be warranted in the future.

## Figures and Tables

**Table 1:** The sequences of q-PCR primers.

Gene	Forward primer	Reverse primer	Size	Number
CCL2	AGCAAGTGTCCCAAAGAAGC	CATGGAATCCTGAACCCACT	93	NM_002982
CCL5	ACCACTCCCTGCTGCTTTG	ACACTTGGCGGTTCCTTCG	212	NM_001278736
CCL8	ATGCTGAAGCTCACACCCTT	TCAAGCTCTGACTCTCAGTCCA	363	NM_005623
CCL15	ATATAATAATAAAGAGACAAAAGAGGC	TACTCTTTATTAGATGCATTACTTTCA	134	NM_032965
CCL19	AGCTCCTCTGCACCAGACCT	TAGTTGTAAACACCAGGCGG	150	NM_006274
CCL21	GATGCAGCGTCTGGACAA	TTGGAGCCCTTTCCCTTC	102	NM_002989.3
CCL23	TGTGTCCAGCTTCAGCATTC	TTTGAAACGAACAGCGAGTG	126	NM_145898
CCL28	AGAAGCCATACTTCCCATTGC	AGCTTGCACTTTCATCCACTG	208	NM_148672
CXCL1	AGGGAATTCACCCCAAGAAC	CACCAGTGAGCTTCCTCCTC	204	NM_001511
CXCL2	CTGCCCTTACAGGAACAGAA	ATCAGGATTGAACTAACTTGGG	250	NM_002089
CXCL8	ACCGGAAGGAACCATCTCACT	ATCAGGAAGGCTGCCAAGAG	75	NM_000584
CXCL9	ATTGGTGCCCAGTTAGCC	CATCAGCAGTGTGAGCAGTG	143	NM_002416
CXCL10	GCTGCTACTACTCCTGTAGGAAGG	TGGAAGATGGGAAAGGTGAG	159	NM_001565
CXCL11	ATGAGTGTGAAGGGCATGGC	TCACTGCTTTTACCCCAGGG	121	NM_005409
CCR1	TCAACAAAGTCACCCACTTCC	GTGTCTCCCATGGCTTAGGA	106	NM_001295
CCR3	TGACTGTGAGCGGAGC	ATGTATCTGCCCAGGTGC	171	NM_178329
CCR5	GACTCTTGGGATGACGC	GATCGGGTGTAAACTGAGC	177	NM_000579
CXCR2	ACATTCCAAGCCTCATGTCC	CTTAGAACATAGAGTGCCATGGG	217	NM_001168298
CXCR4	ACGTAAAGCTAGAAATGATCCCC	GTACACTGTAGGTGCTGAAATCAAC	190	NM_003467
